# Reporting Quality and Risk of Bias Analysis of Published RCTs Assessing Anti-CGRP Monoclonal Antibodies in Migraine Prophylaxis: A Systematic Review

**DOI:** 10.3390/jcm13071964

**Published:** 2024-03-28

**Authors:** Dimitrios Rikos, Michail Vikelis, Emmanouil V. Dermitzakis, Panagiotis Soldatos, Dimitrios Rallis, Jobst Rudolf, Anna P. Andreou, Andreas A. Argyriou

**Affiliations:** 1 404 Military Hospital, 41222 Larisa, Greece; 2 Headache Clinic, Mediterraneo Hospital, 16675 Athens, Greece; mvikelis@headaches.gr; 3 Euromedica General Clinic, 54645 Thessaloniki, Greece; manolis.dermitzakis@gmail.com; 4 Independent Researcher, 24100 Kalamata, Greece; soldatosp@gmail.com; 5 Department of Neurology, Tzaneio General Hospital of Piraeus, 18536 Athens, Greece; jimrallis@hotmail.com; 6 Department of Neurology, Papageorgiou General Hospital of Thessaloniki, 54645 Thessaloniki, Greece; jobstrudolf@hotmail.com; 7 Headache Centre, Guy’s and St Thomas’ NHS Foundation Trust, London SE1 9RT, UK; anna.andreou@headache-research.com; 8 Headache Research-Wolfson Sensory, Pain and Regeneration Centre, Institute of Psychiatry, Psychology and Neuroscience, King’s College London, London SE1 1LU, UK; 9 Headache Outpatient Clinic, Department of Neurology, Agios Andreas General Hospital of Patras, 26335 Patras, Greece; andargyriou@yahoo.gr

**Keywords:** migraine, prevention, calcitonin gene-related peptide (CGRP), monoclonal antibody, quality, risk of bias, randomized clinical trials, erenumab, fremanezumab, galcanezumab, eptinezumab

## Abstract

**Objective:** Phase II/III randomized clinical trials (RCTs) are vulnerable to many types of bias beyond randomization. Insights into the reporting quality of RCTs involving migraine patients treated with monoclonal antibodies targeting the calcitonin gene-related peptide system (anti-CGRP MAbs) are currently lacking. Our aim was to analyze the reporting quality of phase II/III RCTs involving migraine patients treated with anti-CGRP MAbs. **Methods:** A systematic search was performed on the PubMed and EMBASE databases, according to PRISMA guidelines, for relevant RCTs in either episodic or chronic migraine prevention. Additionally, an adapted version of the 2010 CONSORT statement checklist was utilized. The ROBvis online tool was used to document the risk of bias. **Results:** From the initially identified 179 articles, we finally found 31 RCTs that were eligible for evaluation. The average CONSORT compliance was 88.7% (69.7–100%), while 93.5% (N = 29) of the articles had a compliance greater than 75%. Twenty-eight CONSORT items were reported in more than 75% of the articles. The average compliance of the analyzed RCTs was 93.9% for Galcanezumab, 91.3% for Fremanezumab, followed by 85.4% for Erenumab and Eptinezumab studies. Implementation of the ROB2 tool showed some concerning “missing information” arising from the inadequate reporting. Specifically, 50% of the studies (N = 16) were categorized as having inadequate information regarding the randomization process. **Conclusions:** Adequate reporting quality was disclosed in the evaluated RCTs with anti-CGRP MAbs in migraine prevention. However, some methodological issues need to be highlighted to be addressed in future studies assessing the efficacy of new molecules targeting CGRP or other candidate pathways implicated in migraine pathophysiology.

## 1. Introduction

Migraines, a common neurological disorder with a prevalence of about 15% in the general population of Western countries, pose a significant burden on the quality of life of affected patients [1,2]. Generally, migraines are characterized by recurrent moderate or severe headaches and other accompanying symptoms, preceding, overlapping, or enduring within the evolving prodromal, aura phase, ictal, and postictal phases [3,4]. However, most burdensome, even more than the headache itself for some patients, are the hypersensitivity symptoms that occur to various extents of severity during a migraine attack, including osmophobia, phonophobia, photophobia, nausea/vomiting, and cutaneous allodynia [5,6].

According to the frequency of mean monthly headache days (MHDs), episodic migraines are classified, based on the 2018 criteria of the third edition of the International Classification of Headaches Disorders (ICHD3) [7], into very low (1–3 MHDs) (EM), low-frequency (4–7 MHDs), and high-frequency (8–14 MHDs) episodic migraines (HFEMs). Furthermore, patients having headaches occurring on at least 15 days per month, with 8 of these having typical migrainous features for more than 3 months, are diagnosed with chronic migraines (CMs). Although CMs are much more disabling and are associated with substantially less general productivity than episodic migraines, due their its much more severe and complex clinical phenotype, coupled with the increased incidence of comorbidities—mainly psychiatric ones [8,9]—there is evidence to demonstrate that the associated disability of patients with HFEMs is comparable to that of CM patients with 15–23 MHDs [10].

Treatment of migraines consists of acute and preventive approaches. Conventional acute treatments include analgesics, non-steroidal anti-inflammatory agents, and triptans. Preventive oral medications include antiepileptics (topiramate and valproate), antidepressants (venlafaxine, duloxetine amitriptyline), b-blockers (propranolol), and calcium channel blockers such as flunarizine. However, there are significant unmet clinical needs concerning both acute and preventive migraine therapies, such as a lack of efficacy, poor tolerability [11], a higher likelihood of harm than to help, and a lack of persistence, particularly with oral preventives [12,13].

In recent years, the identification of calcitonin gene-related peptide (CGRP) as a critical contributor in migraine pathophysiology [14] represented a game changer, as further revolutionary research findings led to the development of a new class of injectable migraine preventives called the anti-CGRP monoclonal antibodies (anti-CGRP MAbs) [15]. Thus far, their efficacy and safety have been robustly demonstrated in their corresponding pivotal phase II/III clinical studies [16,17,18,19,20]. High-quality randomized clinical trials (RCTs) play a pivotal role in medicine, serving as the gold standard for evaluating the safety and efficacy of medical interventions [21].

Applying the RCT methodology, though, may be challenging. Several potential sources of bias can arise, which may impact the validity and reliability of the study results. Some common sources of bias in RCTs include selection bias, performance bias, attrition bias, baseline imbalance, reporting bias, and others. The latter may conceal several other sources of bias in the case of vague reporting of important methodological aspects.

To tackle such reporting bias, the Consolidated Standards of Reporting Trials (CONSORT) were published and last updated in 2010 [22]. This statement consists of several evidence-based sets of recommendations, including a checklist and a flow diagram for the reporting of RCTs, thus promoting transparent and thorough reporting. The recommendations concern all aspects of the trial, from the title, the methodology, the analysis, the results, the discussion, and, if reported, it may reflect the true trial plan and conduct.

The literature contains several publications involving systematic reviews and meta-analyses concerning the efficacy and safety of anti-CGRP MAbs based on RCTs [23,24,25,26]. However, there is only scarce evidence concerning the critical evaluation of the methodological quality of these RCTs. There are only a few available review studies systematically assessing the methodology of real-world studies with anti-CGRP MAbs for the preventive treatment of migraine [27] or critically discussing the outcomes and endpoints in preventive migraine RCTs [28,29]. Hence, in the current study, we systematically analyzed, according to PRISMA guidelines, the reporting quality of phase II/III RCTs involving migraine patients treated with anti-CGRP MAbs using an adapted version of the 2010 CONSORT statement checklist, thus providing insight into the publicly available information regarding these trials and their potential sources of bias. We also aimed to offer practical recommendations regarding the use of anti-CGRP MAbs in the setting of migraine prevention.

### 1.1. Mechanistic Background for Targeting CGRP to Achieve Migraine Prophylaxis

CGRP exists in two isoforms in the human body, i.e., α-CGRP and β-CGRP, which are derived from separate genes and differ by three amino acids. The 37-amino-acid neuropeptide α-CGRP is the main form expressed in trigeminal ganglia neurons and the form that is most essential to migraine pathology. In contrast, β-CGRP is primarily expressed in enteric nerves and the pituitary gland [30]. The CGRP receptor is formed by a multimer of a G-protein-coupled receptor called calcitonin-like receptor (CLR), the receptor component protein (RCΡ), and a transmembrane protein called receptor activity-modifying protein 1 (RAMP1) [31]. The CLR requires the RAMP1 for the binding of CGRP, and both of these components form the binding pocket, while the RCP is required for coupling the CGRP receptor to various intracellular signal transduction pathways [32,33].

CGRP is a peptide with potent general arterial vasodilator abilities that is widely expressed in various systems of the human body, including, among others, the cardiovascular system, where it is considered to have a protective role [34]. Meanwhile, CGRP and its receptors are also widely located in both the central (CNS) and peripheral nervous system (PNS), including the trigeminal ganglion and the trigeminovascular pathways [35]. CGRP is located in about 50% of neurons and in unmyelinated C fibers, whereas elements of the CGRP receptor are expressed in about 40% of the neurons within the trigeminal ganglion and in myelinated A-fibers. Notably, these elements form the connection between the PNS and the CNS, thoroughly bolstering the importance of CGRP in maintaining the integrity of sensory and motor functions between central and peripheral nerve structures [33]. The release of CGRP at the peripheral synapses of trigeminal terminals results in vasodilation through binding of CGRP to its receptors at the level of the smooth muscle cells of meningeal and cerebral blood vessels [36].

Accumulating evidence suggests that CGRP ranks among the most important contributors in migraine pathogenesis, as demonstrated by its release upon stimulation of the trigeminal ganglion [37] coupled with evidence of CGRP-related modulation of the transmission of pain signals from the meninges to the CNS [31]. On clinical grounds, administering CGRP in migraineurs can evoke acute headaches, while inversely, CGRP release is suppressed by acute anti-migraine agents such as triptans. There is evidence of significantly and persistently elevated CGRP levels in patients with chronic migraines [32,38]. Furthermore, CGRP levels were significantly reduced in subjects who responded to sumatriptan treatment after an NO_2_-induced migraine, while there was no significant reduction in CGRP levels in sumatriptan non-responders [39]. As such, CGRP, via its pain modulation abilities, appears to be an important therapeutic target in migraine prophylaxis, as evidenced in numerous preclinical and clinical studies [40].

### 1.2. Anti-CGRP/CGRPr MAbs for Migraine Prophylaxis

Currently, four MAbs targeting CGRP or its receptor (CGRPr) are commercially available for migraine prevention. The MAbs that have been studied and formally approved for use in episodic and chronic migraine prophylaxis are Erenumab, which targets the CGRPr, and Fremanezumab, Galcanezumab, and Eptinezumab, which target the CGRP ligand. Due to their large size, with molecular weights between ~143 and 150 kDa, none of these four antiCGRP/CGRPr Mabs can cross the blood–brain barrier and act from the periphery [41].

#### 1.2.1. Erenumab

Erenumab is a human monoclonal antibody with a molecular weight of ~150 kDa, containing 1344 amino acids. It binds with a high affinity to the canonical CGRPr receptor and antagonizes CGRPr function to provide migraine prevention to both EM and CM patients through blockage of both the CGRP and CLR/RAMP1 receptors within the trigeminal system. Furthermore, erenumab is able to eventually evoke alterations in the CGRP signaling pathway, coupled with reduced production of CGRP-induced cAMP [42].

#### 1.2.2. Fremanezumab

Fremanezumab is a large-size (molecular weight ~150 kDa), fully humanized IgG 2(delta) a/kappa monoclonal antibody. It has approval to be commenced as a prophylactic treatment for both EM and CM based on its ability to selectively inhibit the activation of the trigeminovascular pain pathway. Its mode of action mainly comprises potently and selectively binding with a high affinity to both α- and β-CGRP isoforms to prevent them from attachment to the CGRP receptor in the trigeminal ganglion and meningeal nociceptors [43].

#### 1.2.3. Galcanezumab

Galcanezumab, a humanized monoclonal antibody (molecular weight of ~147 kDa) produced in Chinese Hamster Ovary cells with the use of recombinant DNA technology, was clinically developed for EM and CM prophylaxis by blocking the CGRP pathway and the subsequent downstream activation of the intracellular signaling pathways via targeting of the CGRP ligand itself. Galcanezumab blocks CGRP with a high affinity (KD = 31 pM) and high specificity (>10,000-fold vs. related peptides adrenomedullin, amylin, calcitonin, and intermedin), thus preventing its biological activity [44].

#### 1.2.4. Eptinezumab

Eptinezumab, a fully humanized antibody (molecular weight of ~143 kDa) manufactured using yeast (*Pichia pastoris*), is approved for the preventive treatment of episodic and chronic migraines in adults. Its mode of action is mainly based on its ability to selectively bind to both α- and β-CGRP isoforms of the human CGRP to immediately inhibit their attachment to the CGRP receptor as a result of 100% bioavailability via its intravenous route of administration [45]. As such, it is considered that eptinezumab associates rapidly and dissociates more slowly from the CGRP compared to other MAbs targeting the CGRP ligand, i.e., fremanezumab and galcanezumab [46]. Table 1 summarizes the main characteristics of commercially available anti-CGRP MAbs for migraine prophylaxis.

## 2. Methods

### 2.1. Search Strategy and Eligibility of Studies

This systematic review was performed in accordance to the PRISMA guidelines. We performed a systematic search on the PubMed and EMBASE databases until the 1st of October 2023 for phase II/III RCTs concerning the use of anti-CGRP MAbs in either EM or CM prophylaxis. We used a combination of keywords including “erenumab (Novartis AG, Basel, Switzerland)”, “fremanezumab (Teva Pharmaceutical Company, Tel Aviv, Israel)”, “galcanezumab (Eli Lilly and Company, Cambridge, MA, USA)”, “eptinezumab (Lundbeck A/S, Copenhagen, Denmark)”, “AMG334”, “TEV-48125”, “ALD403”, and “LY2951742”, and we used “RCTs” as a filter.

Trials were eligible for inclusion if the participants were humans and had been randomly assigned to at least two medicinal treatment arms. Studies should be reported in English in full and regard the main analysis of an RCT. Hence, secondary, post-hoc, extension, or pooled analysis results were excluded. Studies regarding non-migraine use of anti-CGRP mAbs were also excluded. After the initial search, one researcher (D.Ri) excluded irrelevant studies. The rest were assessed for eligibility by two researchers (A.A.A. and E.V.D.) independently, and any discrepancies were resolved by consensus.

### 2.2. Data Extraction and Implementation of Assessment Tools

From each eligible article, the basic information was extracted (author, year, compound studied), while the reporting assessment was carried out through the implementation of the Consolidated Standards of Reporting Trials (CONSORT) 2010 checklist [22]. The checklist consists of 37 questions organized in five sections regarding the basic elements of an RCT article (title, introduction, methods, results, and funding). In this study, we used an adaptation of the checklist by removing 4 questions (3b, 6b, 7b, 14b) which usually correspond to not applicable situations/conditions.

Two investigators independently assessed each eligible article and assigned a positive or negative (“yes” or “no” format) response to each CONSORT item based on whether it was reported or not. If a procedure was actually carried out during the trial but there was no lucid reference in the article, it was considered not to have been reported. For example, if a trial stated that the study was a “multicenter, double-blind, controlled trial” but there was no word “randomized” in the title, the respective CONSORT item (1a) was assigned a negative (“no”) response. The evaluation was carried out by two independent authors (D.Ra. and P.S), and any discrepancies were resolved by consensus.

The quantification of the reporting quality of the articles was performed by calculating the “CONSORT compliance”, meaning the percentage of the 33 CONSORT items that each article addressed. Descriptives of the compliance, including more than 75% compliance, and the total number of CONSORT questions reported in more than 75% of the articles are reported, following other similar articles [47,48,49]. Comparisons among different drug RCTs and by year of publication are also explored. All statistical analyses were carried out using either Microsoft Excel 2019 (Microsoft Corporation, Redmond, WA, USA) or SPSS for Windows (release 27.0; SPSS Inc., Chicago, IL, USA) software.

The Cochrane risk-of-bias tool for randomized trials (ROB2) [50] was used to profile the potential bias emerging from the RCTs reporting, as was recorded at the previous step. ROB2 assesses the risk of bias in five domains (domain 1: risk of bias arising from the randomization process; domain 2: risk of bias due to deviations from the intended interventions (effect of assignment to intervention); domain 3: missing outcome data; domain 4: risk of bias in the measurement of the outcome; domain 5: risk of bias in the selection of the reported result). Each time inadequate reporting was detected, a “No information” response was added to the risk-of-bias tool. The responses “Yes, probably Yes, No, probably No” were not used. This was because we cannot be sure whether something was actually carried out, as we did not reach for the RCTs’ actual results but the only for the published ones. A study with missing information in more than two domains was categorized as a “No information” study. The ROBvis online tool (https://www.riskofbias.info/welcome/robvis-visualization-tool, accessed on 1 November 2023) [51] was used for the visualization of the ROB2 documented risk of bias.

## 3. Results

**Figure 1:**
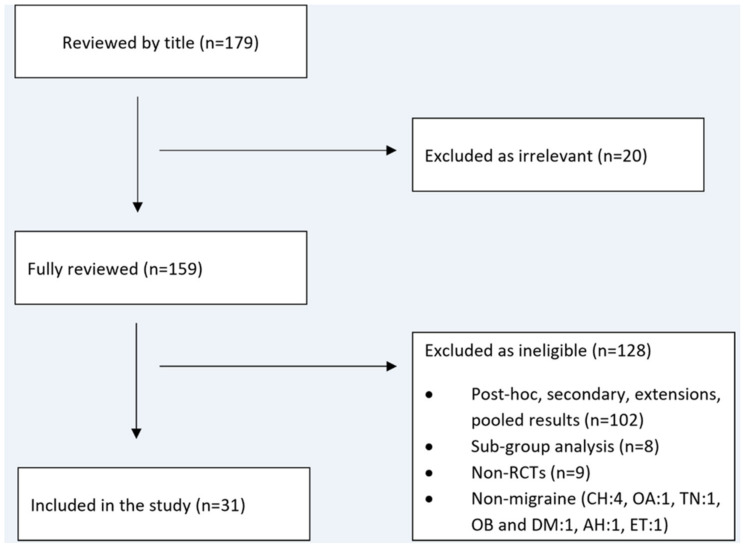
Flow diagram of our systematic search. The abbreviation CH stands for cluster headache; OB and DM for obesity and diabetes mellitus; OA for osteoarthritis; TN for trigeminal neuralgia; AH for arterial hypertension; and ET for essential tremor.

We initially identified 179 articles. By implementing the eligibility criteria, 20 articles were excluded as irrelevant, and the remaining 159 were fully reviewed by two investigators (A.A.A and E.V.D). The second step resulted in the exclusion of 128 articles as ineligible, thus leading to the final inclusion of 31 RCTs [16,17,18,19,20,52,53,54,55,56,57,58,59,60,61,62,63,64,65,66,67,68,69,70,71,72,73,74,75,76,77]. Figure 1 shows a flow diagram of our systematic search, according to the PRISMA guidelines.

The 31 RCTs were published in 10 different scientific journals from 2014 to 2022 and included a total of 20,063 patients (average 647 patients). The smallest trial enrolled 174 patients [56] and the largest enrolled 1696 patients [70]. As can be seen in Table 2, 11 studies concerned erenumab [20,55,59,60,65,66,69,73,74,75,77]; 7 galcanezumab [17,57,62,64,70,71,72]; 7 fremanezumab [18,19,53,54,61,67,68] and 6 eptinezumab [16,52,56,58,63,76].

The majority of the studies reported a double-blind period of 12 weeks (N = 21, 70.1%), while fewer studies had a double-blind period of 24 weeks (N = 7, 22.51%) [52,60,65,69,70,72,74]. The study by Goadsby et al. [61] evaluated the long-term safety, tolerability, and efficacy of fremanezumab over a double-blind period of 52 weeks. One study [76] evaluated the effects of intravenous eptinezumab on headache pain and the most bothersome symptom when initiated during a migraine attack. In this study, the evaluation timeframe was the time to freedom from headache pain; thus, it was the smallest of all the studies. Most of the studies (N = 24, 72.7%) used the change from baseline in monthly migraine days (MMD) as their primary endpoint. The studies by Dodick et al. [58] and Reuter et al. [66] used the percentage of patients achieving a 75% and 50% reduction in monthly migraine days, respectively. In the study of Bigal et al., regarding the use of fremanezumab in chronic migraines (CMs), the authors used the change from baseline in the number of headache hours during the third treatment cycle (weeks 9–12) as their primary endpoint [54]. Two studies [56,61] had as a primary goal the assessment of safety after infusion of eptinezimab and fremanezumab, respectively. The only study that used an active drug as a comparator was the one by Reuter et al. [65], in which the primary endpoint was the number of patients that discontinued medication due to an adverse event. Another novel study design in the anti-CGRP mAbs research landscape was the study by Winner et al. in which the investigative drug (Eptinezumab) was initiated during a migraine attack and the primary endpoint was the time since freedom from pain [76].

The average CONSORT compliance was 88.7% (69.7–100%), while 93.5% (N = 29) of the articles had a compliance greater than 75%. Twenty-eight CONSORT items were reported in more than 75% of the articles, while item 10 (“Who generated the random allocation sequence, who enrolled participants, and who assigned participants to interventions”) was the least addressed, as it was reported in just 13 articles [53,54,57,65,66,67,68,69,71,73,74,75,77] (Table 3).

**Figure 2:**
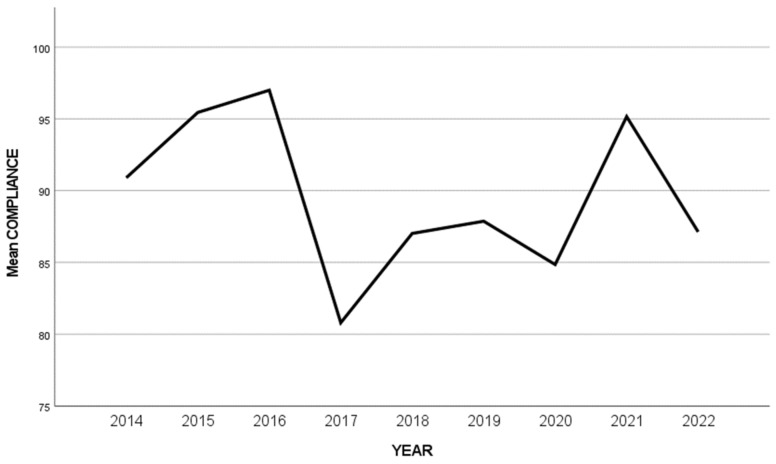
Plot of the average CONSORT compliance per year.

The average compliance of the analyzed RCTs was 93.9% for galcanezumab, 91.3% for fremanezumab, followed by 85.4% for the erenumab and eptinezumab studies (Table 2). The study by Takeshima et al. [74] was the only study that achieved the highest CONSORT compliance possible (100%). This study was a randomized, double-blind, placebo-controlled study assessing the efficacy and safety of erenumab for migraine prevention in Japanese patients. On the other hand, the smallest compliance percentage was achieved by a study by Ashina et al. (69.7%), mostly due to inadequate reporting of the randomization process. This study was a randomized, double-blind, placebo-controlled study of eptinezumab in episodic migraines [16]. The reporting quality significantly differed among the years (2014 to 2023), but with no apparent reason or trend of increasing quality (Figure 2).

**Figure 3:**
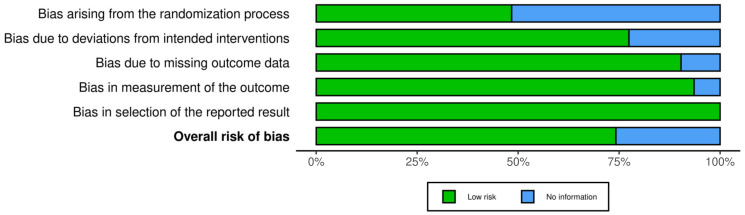
Impact of poor reporting quality on the five domains of potential bias.

**Figure 4:**
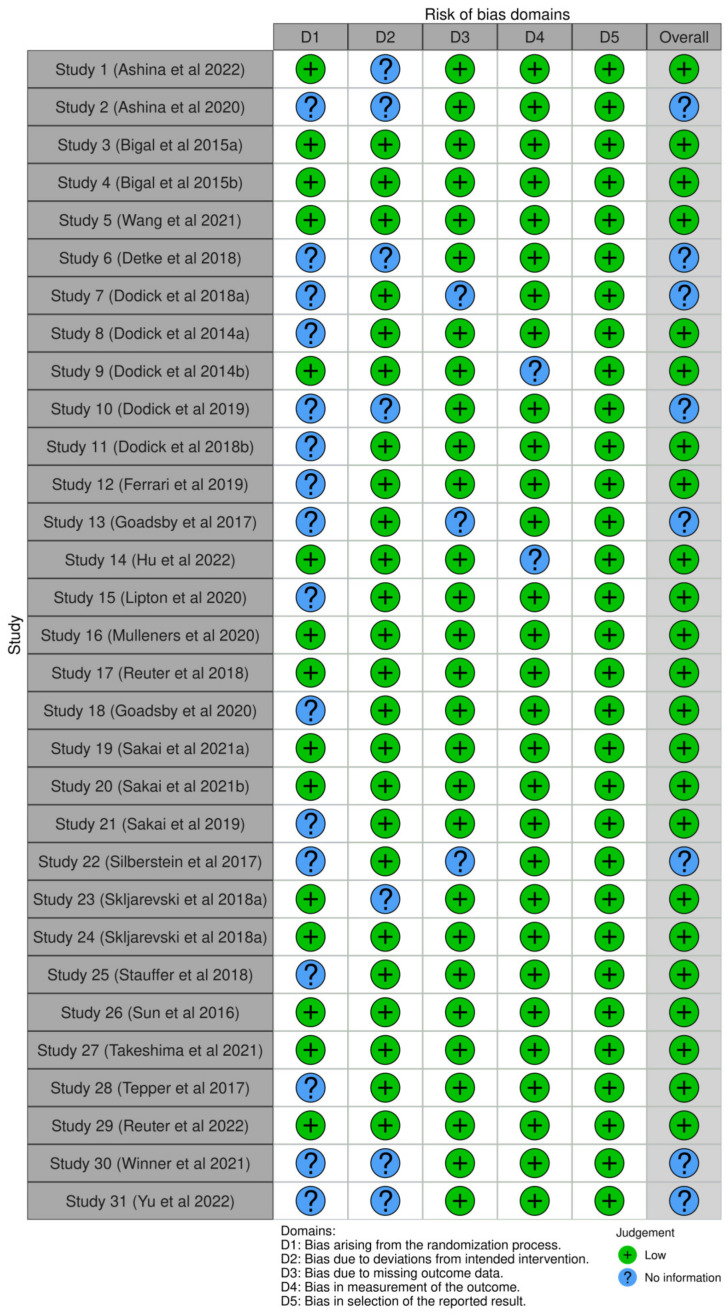
Traffic light plot of the impact of reporting on each study’s potential bias assessment [16,17,18,19,20,52,53,54,55,56,57,58,59,60,61,62,63,64,65,66,67,68,69,70,71,72,73,74,75,76,77].

Implementation of the ROB2 tool showed some concerning “missing information” arising from the inadequate reporting. Specifically, 50% of the studies (N = 16) were categorized as having inadequate information regarding the randomization process [16,17,18,19,20,55,56,58,59,60,61,63,69,72,76,77]. This regards the first domain of the ROB2 tool, which includes three basic questions: (1) “Was the allocation sequence random?”; (2) “Was the allocation sequence concealed until the participants were enrolled and assigned to interventions?”; and (3) “Did baseline differences between intervention groups suggest a problem with the randomization process?” [50]. This is conveniently visualized by the ROB-vis toll (Figure 3 and Figure 4).

## 4. Discussion

The elucidation of the role of CGRP in migraine pathophysiology resulted in the discovery and clinical development of new therapeutic avenues in the management of patients with migraines; mostly in the setting of migraine prevention. Based on their mechanism of action, targeting either the CGRP or the CGRPr, four anti-CGRP MAbs have been approved for clinical use in migraine prevention by the regulatory authorities based on compelling results of RCTs to support an excellent efficacy/safety profile [78].

Although systematic reviews and meta-analyses may contribute to medical decision-making, RCTs are their structural components; therefore, their quality is crucial. RCTs’ design allows them to systematically compare treatments, interventions, or drugs under controlled conditions, aiming to remove any other between-group differences except for the intervention itself, thus providing robust evidence reflecting its true effects [21]. As such, the thorough reporting of methodology within RCTs is definitely of paramount importance in conducting evidence-based medical research [79].

Conducting methodologically robust RCTs represents the cornerstone starting point before any medication obtains approval from the regulatory agencies to enter into everyday clinical practice. RCTs’ design allows them to systematically compare treatments, interventions, or drugs under controlled conditions, aiming to remove any other between-group difference except the intervention itself and thus providing robust evidence reflecting its true effects [21]. We herein systematically searched for those RCTs regarding patients treated with anti-CGRP MAbs for their migraine prophylaxis and reported on their methodological quality. A total of 31 RCTs published in 10 top-ranked journals and involving 20,063 patients were traced. MMDs rather than MHDs were the most commonly used primary outcome, and as such, all commercially available anti-CGRP MAbs gained regulatory approval using this metric.

This study used the CONSORT statement in an inverted manner, and it seems that the evaluated RCTs with anti-CGRP MAbs in the prevention of migraine have adequate reporting quality. However, there are some issues that emerged and need to be addressed. The average compliance with the assessment tool was 88.7%, which is compelling compared to other neurological conditions that have similarly been previously assessed such as restless leg syndrome (1998–2016): 56.5% [47] or multiple sclerosis (2000–2015: 68.2% [48]).

Furthermore, there are important items that have been reported by all the RCTs included. These are items 1b, 2a, 6a, 8b, 12a, 12b, 15, 17a, 17b, 18, 19, 22, and 23 (Table 3), which concern adequate reporting of statistical methods used and having predefined outcomes. Furthermore, all the articles reported their trial’s registration number, as opposed to previous years where this reporting was not generally applicable due to the Clinical Trials Registration webpage (clinicaltrials.gov) being launched in 2000 and currently achieving universal use in the scientific research community.

On the other hand, there are important items that were significantly underreported. The most important are items 10 and 9 of the CONSORT 2010 checklist, which were the least reported (41.9% and 48.4%, respectively). Item 10 (“Who generated the random allocation sequence, who enrolled participants, and who assigned participants to interventions”), along with item 9 (“Mechanism used to implement the random allocation sequence (such as sequentially numbered containers), describing any steps taken to conceal the sequence until interventions were assigned”), refer to the randomization process and implementation.

Clearly defining the sample size calculations, the randomization process, and the clinical endpoints are important elements of RCTs to help produce reliable results and to heighten the interpretability and generalizability of the results. We identified 31 RCTs involving 20,063 patients, with an average of 647 enrolled patients per trial, to thoroughly support the adequacy of the sample sizes studied. However, this is not completely true if we consider that the number of enrolled patients greatly varied between the RCTs, from 174 patients in the weaker trial from a sample size perspective [56] to almost 1700 patients in the most sufficiently powered study [70]. As such, sample size estimations are definitely needed to pre-define the number of enrolled patients according to the RCTs’ arms.

According to our analysis, MMDs were mostly (N = 24/21 RCTs; 72.7%) used as a primary outcome to quantify the efficacy of anti-CGRP MAbs in preventing migraines and to eventually gain their approval. Other RCTs used other metrics, such as the MHDs, the change from baseline in the number of headache hours during the third treatment cycle (weeks 9–12) [54], or head-to-head comparisons with standard of care [65]. Nonetheless, it seems that MMDs are generally preferred to measure the efficacy of anti-CGRP MAbs in line with established guidelines [80], although there is no evidence to clearly demonstrate the superiority of this metric over MHDs due to a lack of consensus concerning the definition of a migraine day [81]. Furthermore, in terms of cost-effectiveness, the frequency distribution of MHDs and MMDs is of comparable importance to establishing the efficacy of migraine prevention [82], while both of these metrics were used in RCTs [28]. Tackling this inconsistency to allow for reliable comparison of results is needed, and as such, further RCTs should provide the readers with clear definitions of migraine days and headache days and determine if the 50% or 30% threshold in the reduction of migraine frequency is set as an endpoint. Currently, both of these thresholds are accepted in current guidelines [83], although it remains unclear in our opinion whether a 30% responder rate is indeed enough for moderately/highly disabled migraine patients.

We also demonstrate that issues with randomization reporting increase the risk of bias in preventive migraine clinical trials. Considering that randomization is one of the most significant procedures for conducting top-level clinical research, it is suggested that any doubts regarding the integrity and performance of good clinical practice related to randomization issues are juddering the framework of RCTs and, as such, should be thoroughly omitted.

We have to acknowledge some limitations of our study, including the following: i) the interpretation of methodological analysis for the included RCTs might be susceptible to subjectivity, as the evaluators were not blinded to journal. Additionally, certain items like flow diagrams, randomization, and blinding could likely have gained more focus than others. ii) We used an adapted version of the 2010 CONSORT checklist for all RCTs, regardless of their publication date. However, this checklist per se was constructed to guide researchers on how to perform RCTs rather than being used as an evaluation tool to critically assess the quality of the evidence provided. As our analysis was limited to core phase II/III RCTs as a primary objective, no inferences on other forms of RCTs (e.g., results of secondary, post-hoc, extension or pooled analyses) can be made.

## 5. Conclusions

Our findings confirm that, indeed, anti-CGRP MAbs have opened new therapeutic horizons in migraine prevention based on the corresponding RCTs, which all had an overall adequate methodological quality and predefined outcomes. In this study, we further reveal some issues that require attention to reduce the risk of bias and improve the quality of reporting for future RCTs in migraine prevention.

On the general grounds of potential improvements in reporting quality in future RCTs of anti-migraine preventative treatments, we urge the need for standardization of protocol procedures, randomization and consistent endpoint selection, as well as the use of universal definitions of migraine/headache days to minimize the risk of bias. Such a strategy would enhance the ability to make direct comparisons and informed decisions among these medications based on evidence-based medicine. Furthermore, consistency in patients’ self-reported outcome measures is warranted to reliably capture other important measures, such as changes in disability, impairment, and quality of life status.

## Figures and Tables

**Table 1:** Summary of the main characteristics of commercially available anti-CGRP MAbs for migraine prophylaxis.

INN or Code Name	AMG334/Erenumab	TEV-48125/Fremanezumab	LY2951742/Galcanezumab	ALD-403/Eptinezimab
Brand name	Aimovig^TM^	Ajovy^TM^	Emgality^TM^	Vyepti^TM^
Molecular format	Human IgG_2_ (100% human)	Humanized IgG_2_ (>95% human)	Humanized IgG_4_ (>95% human)	Humanized IgG_1_ (>90% human)
Expression system	Chinese hamster ovary	Murine	Murine	Yeast
Target	CGRPr	CGRP	CGRP	CGRP
Administration	Subcutaneous	Subcutaneous	Subcutaneous	Intravenous
Dosing	70–140 mg every 28 days	225 mg monthly or 3 × 225 mg quarterly	120 mg monthly (240 mg starting dose)	100–300 mg every 12 weeks
Half-life time	21 days	31 days	~25–30 days	~30 days
Indication	Migraine prevention in adults experiencing more than 4 migraine days monthly			
Time of EU approval	07/2018	02/2019	11/2018	01/2022

**Table 2:** Descriptives of RCTs, reporting methodological quality, by each anti-CGRP MAb studied.

	Erenumab (N = 11)	Galcanezumab (N = 7)	Fremanezumab (N = 7)	Eptinezumab (N = 6)
Average Compliance % (min–max)	85.4 72.7–97	93.9 87.9–100	91.3 78.8–97	85.4 69.7–97
Publication years	2016–2022	2014–2022	2015–2021	2014–2022
Randomized patients (total, min, max)	6736 246–955	5286 218–1696	3806 264–1130	4235 174–1121

**Table 3:** Average compliance of each CONSORT item.

Item	Average Compliance
Abstract/Title	
1a	90.3
1b	100.0
Introduction	
2a	100.0
2b	96.8
Methods	
3a	90.3
4a	96.8
4b	54.8
5	90.3
6a	100.0
7a	90.3
8a	90.3
8b	100.0
9	48.4
10	41.9
11a	77.4
11b	61.3
12a	100.0
12b	100.0
Results	
13a	90.3
13b	90.3
14a	90.3
15	100.0
16	93.5
17a	100.0
17b	100.0
18	100.0
19	100.0
Discussion	
20	93.5
21	93.5
22	100.0
Other information	
23	100.0
24	48.4
25	96.8

## Data Availability

The data that support the findings of this study are available from the corresponding author upon reasonable request.

## References

[1] Ferrari M.D., Goadsby P.J., Burstein R., Kurth T., Ayata C., Charles A., Ashina M., van den Maagdenberg A.M.J.M., Dodick D.W. (2022). Migraine. Nat. Rev. Dis. Primers.

[2] Steiner T.J., Stovner L.J., Jensen R., Uluduz D., Katsarava Z., Lifting The Burden: The Global Campaign against Headache (2020). Migraine remains second among the world’s causes of disability, and first among young women: Findings from GBD2019. J. Headache Pain.

[3] Vaghi G., De Icco R., Tassorelli C., Goadsby P.J., Vicente-Herrero T., de la Torre E.R. (2023). Who cares about migraine? Pathways and hurdles in the European region-access to care III. J. Headache Pain.

[4] Lipton R.B., Lanteri-Minet M., Leroux E., Manack Adams A., Contreras-De Lama J., Reed M.L., Fanning K.M., Buse D.C. (2023). Pre- and post-headache phases of migraine: Multi-country results from the CaMEO–International Study. J. Headache Pain.

[5] Quintela E., Castillo J., Munoz P., Pascual J. (2006). Premonitory and resolution symptoms in migraine: A prospective study in 100 unselected patients. Cephalalgia.

[6] Charbit A.R., Akerman S., Goadsby P.J. (2010). Dopamine: What’s new in migraine?. Curr. Opin. Neurol..

[7] Arnold M. (2018). Headache classification committee of the international headache society (IHS) the international classification of headache disorders. Cephalalgia.

[8] Buse D.C., Reed M.L., Fanning K.M., Bostic R.C., Lipton R.B. (2020). Demographics, headache features, and comorbidity profiles in relation to headache frequency in people with migraine: Results of the American Migraine Prevalence and Prevention (AMPP) study. Headache J. Head Face Pain.

[9] Blumenfeld A.M., Varon S.F., Wilcox T.K., Buse D.C., Kawata A.K., Manack A., Goadsby P.J., Lipton R.B. (2011). Disability, HRQoL and resource use among chronic and episodic migraineurs: Results from the International Burden of Migraine Study (IBMS). Cephalalgia.

[10] Schwedt T.J., Digre K., Tepper S.J., Spare N.M., Ailani J., Birlea M., Burish M., Mechtler L., Gottschalk C., Quinn A.M. (2020). The American registry for migraine research: Research methods and baseline data for an initial patient cohort. Headache J. Head Face Pain.

[11] Leroux E., Buchanan A., Lombard L., Loo L.S., Bridge D., Rousseau B., Hopwood N., Matthews B.R., Reuter U. (2020). Evaluation of patients with insufficient efficacy and/or tolerability to triptans for the acute treatment of migraine: A systematic literature review. Adv. Ther..

[12] Overeem L.H., Raffaelli B., Mecklenburg J., Kelderman T., Neeb L., Reuter U. (2021). Indirect comparison of topiramate and monoclonal antibodies against CGRP or its receptor for the prophylaxis of episodic migraine: A systematic review with meta-analysis. CNS Drugs.

[13] Jackson J.L., Cogbill E., Santana-Davila R., Eldredge C., Collier W., Gradall A., Sehgal N., Kuester J. (2015). A comparative effectiveness meta-analysis of drugs for the prophylaxis of migraine headache. PLoS ONE.

[14] Edvinsson L., Warfvinge K. (2019). Recognizing the role of CGRP and CGRP receptors in migraine and its treatment. Cephalalgia.

[15] Eigenbrodt A.K., Ashina H., Khan S., Diener H.C., Mitsikostas D.D., Sinclair A.J., Pozo-Rosich P., Martelletti P., Ducros A., Lantéri-Minet M. (2021). Diagnosis and management of migraine in ten steps. Nat. Rev. Neurol..

[16] Ashina M., Saper J., Cady R., Schaeffler B.A., Biondi D.M., Hirman J., Pederson S., Allan B., Smith J. (2020). Eptinezumab in episodic migraine: A randomized, double-blind, placebo-controlled study (PROMISE-1). Cephalalgia.

[17] Detke H.C., Goadsby P.J., Wang S., Friedman D.I., Selzler K.J., Aurora S.K. (2018). Galcanezumab in chronic migraine: The randomized, double-blind, placebo-controlled REGAIN study. Neurology.

[18] Dodick D.W., Silberstein S.D., Bigal M.E., Yeung P.P., Goadsby P.J., Blankenbiller T., Grozinski-Wolff M., Yang R., Ma Y., Aycardi E. (2018). Effect of fremanezumab compared with placebo for prevention of episodic migraine: A randomized clinical trial. JAMA.

[19] Silberstein S.D., Dodick D.W., Bigal M.E., Yeung P.P., Goadsby P.J., Blankenbiller T., Grozinski-Wolff M., Yang R., Ma Y., Aycardi E. (2017). Fremanezumab for the preventive treatment of chronic migraine. N. Engl. J. Med..

[20] Tepper S., Ashina M., Reuter U., Brandes J.L., Doležil D., Silberstein S., Winner P., Leonardi D., Mikol D., Lenz R. (2017). Safety and efficacy of erenumab for preventive treatment of chronic migraine: A randomised, double-blind, placebo-controlled phase 2 trial. Lancet Neurol..

[21] Hariton E., Locascio J.J. (2018). Randomised controlled trials—The gold standard for effectiveness research: Study design: Randomised controlled trials. BJOG Int. J. Obstet. Gynaecol..

[22] Moher D., Hopewell S., Schulz K.F., Montori V., Gøtzsche P.C., Devereaux P.J., Elbourne D., Egger M., Altman D.G. (2010). CONSORT 2010 explanation and elaboration: Updated guidelines for reporting parallel group randomised trials. BMJ.

[23] Yang C.-P., Zeng B.-Y., Chang C.-M., Shih P.-H., Yang C.-C., Tseng P.-T., Wang S.-J. (2021). Comparative effectiveness and tolerability of the pharmacology of monoclonal antibodies targeting the calcitonin gene-related peptide and its receptor for the prevention of chronic migraine: A network meta-analysis of randomized controlled trials. Neurotherapeutics.

[24] Haghdoost F., Puledda F., Garcia-Azorin D., Huessler E.-M., Messina R., Pozo-Rosich P. (2023). Evaluating the efficacy of CGRP mAbs and gepants for the preventive treatment of migraine: A systematic review and network meta-analysis of phase 3 randomised controlled trials. Cephalalgia.

[25] Messina R., Huessler E.-M., Puledda F., Haghdoost F., Lebedeva E.R., Diener H.-C. (2023). Safety and tolerability of monoclonal antibodies targeting the CGRP pathway and gepants in migraine prevention: A systematic review and network meta-analysis. Cephalalgia.

[26] Masoud A.T., Hasan M.T., Sayed A., Edward H.N., Amer A.M., Naga A.E., Elfil M., Alghamdi B.S., Perveen A., Ashraf G.M. (2021). Efficacy of calcitonin gene-related peptide (CGRP) receptor blockers in reducing the number of monthly migraine headache days (MHDs): A network meta-analysis of randomized controlled trials. J. Neurol. Sci..

[27] Vandenbussche N., Pisarek K., Paemeleire K. (2023). Methodological considerations on real-world evidence studies of monoclonal antibodies against the CGRP-pathway for migraine: A systematic review. J. Headache Pain.

[28] McGinley J.S., Houts C.R., Nishida T.K., Buse D.C., Lipton R.B., Goadsby P.J., Dodick D.W., Wirth R.J. (2021). Systematic review of outcomes and endpoints in preventive migraine clinical trials. Headache.

[29] Sharpless L.K., Kesselheim A.S., Orr S.L., Darrow J. (2023). Variation in endpoints in FDA medication approvals: A review of acute and preventive migraine medications. Neurology.

[30] Durham P.L., Vause C.V. (2010). Calcitonin gene-related peptide (CGRP) receptor antagonists in the treatment of migraine. CNS Drugs.

[31] Raddant A.C., Russo A.F. (2011). Calcitonin gene-related peptide in migraine: Intersection of peripheral inflammation and central modulation. Expert Rev. Mol. Med..

[32] Bigal M.E., Walter S., Rapoport A.M. (2013). Calcitonin gene-related peptide (CGRP) and migraine current understanding and state of development. Headache J. Head Face Pain.

[33] Russell F.A., King R., Smillie S.-J., Kodji X., Brain S. (2014). Calcitonin gene-related peptide: Physiology and pathophysiology. Physiol. Rev..

[34] MaassenVanDenBrink A., Meijer J., Villalón C.M., Ferrari M.D. (2016). Wiping out CGRP: Potential cardiovascular risks. Trends Pharmacol. Sci..

[35] Deen M., Correnti E., Kamm K., Kelderman T., Papetti L., Rubio-Beltrán E., Vigneri S., Edvinsson L., Maassen Van Den Brink A. (2017). European Headache Federation School of Advanced Studies (EHF-SAS) Blocking CGRP in migraine patients—A review of pros and cons. J. Headache Pain.

[36] Eftekhari S., Edvinsson L. (2010). Possible sites of action of the new calcitonin gene-related peptide receptor antagonists. Ther. Adv. Neurol. Disord..

[37] Goadsby P.J., Edvinsson L., Ekman R. (1988). Release of vasoactive peptides in the extracerebral circulation of humans and the cat during activation of the trigeminovascular system. Ann. Neurol..

[38] Kaiser E.A., Russo A.F. (2013). CGRP and migraine: Could PACAP play a role too?. Neuropeptides.

[39] Juhasz G., Zsombok T., Jakab B., Nemeth J., Szolcsanyi J., Bagdy G. (2005). Sumatriptan causes parallel decrease in plasma calcitonin gene-related peptide (CGRP) concentration and migraine headache during nitroglycerin induced migraine attack. Cephalalgia.

[40] Charles A. (2018). The pathophysiology of migraine: Implications for clinical management. Lancet Neurol..

[41] Messlinger K. (2018). The big CGRP flood—Sources, sinks and signalling sites in the trigeminovascular system. J. Headache Pain.

[42] Andreou A.P., Fuccaro M., Lambru G. (2020). The role of erenumab in the treatment of migraine. Ther. Adv. Neurol. Disord..

[43] Melo-Carrillo A., Noseda R. (2017). Selective Inhibition of Trigeminovascular Neurons by Fremanezumab: A Humanized Monoclonal Anti-CGRP Antibody. J. Neurosci. Off. J. Soc. Neurosci..

[44] Kielbasa W., Helton D.L. (2019). A new era for migraine: Pharmacokinetic and pharmacodynamic insights into monoclonal antibodies with a focus on galcanezumab, an anti-CGRP antibody. Cephalalgia.

[45] Berger A.A., Keefe J., Stark C.W., Moore M., Ramírez G.F., Cucarola J.R., Han A.H., Kaye A.D., Ganti L. (2022). Eptinezumab-jjmr, a humanized monoclonal specific to Calcitonin Gene Related Peptide, for the preventive treatment of migraine in adults. Health Psychol. Res..

[46] Scuteri D., Adornetto A., Rombolà L., Naturale M.D., Morrone L.A., Bagetta G., Tonin P., Corasaniti M.T. (2019). New trends in migraine pharmacology: Targeting calcitonin gene–related peptide (CGRP) with monoclonal antibodies. Front. Pharmacol..

[47] Rikos D., Dardiotis E., Aloizou A.M., Siokas V., Zintzaras E., Hadjigeorgiou G.M. (2019). Reporting Quality of Randomized Controlled Trials in Restless Legs Syndrome Based on the CONSORT Statement. Tremor Other Hyperkinet. Mov..

[48] Rikos D., Dardiotis E., Tsivgoulis G., Zintzaras E., Hadjigeorgiou G.M. (2016). Reporting quality of randomized-controlled trials in multiple sclerosis from 2000 to 2015, based on CONSORT statement. Mult. Scler. Relat. Disord..

[49] Vrysis C., Beneki E. (2023). Assessment of the reporting quality of randomised controlled trials for vitamin D supplementation in autoimmune thyroid disorders based on the CONSORT statement. Endocrine.

[50] Sterne J.A.C., Savović J., Page M.J., Elbers R.G., Blencowe N.S., Boutron I., Cates C.J., Cheng H.Y., Corbett M.S., Eldridge S.M. (2019). RoB 2: A revised tool for assessing risk of bias in randomised trials. BMJ.

[51] McGuinness L.A., Higgins J.P.T. (2020). Risk-of-bias VISualization (robvis): An R package and Shiny web app for visualizing risk-of-bias assessments. Res. Syn. Meth..

[52] Ashina M., Lanteri-Minet M., Pozo-Rosich P., Ettrup A., Christoffersen C.L., Josiassen M.K., Phul R., Sperling B. (2022). Safety and efficacy of eptinezumab for migraine prevention in patients with two-to-four previous preventive treatment failures (DELIVER): A multi-arm, randomised, double-blind, placebo-controlled, phase 3b trial. Lancet Neurol..

[53] Bigal M.E., Dodick D.W., Rapoport A.M., Silberstein S.D., Ma Y., Yang R., Loupe P.S., Burstein R., Newman L.C., Lipton R.B. (2015). Safety, tolerability, and efficacy of TEV-48125 for preventive treatment of high-frequency episodic migraine: A multicentre, randomised, double-blind, placebo-controlled, phase 2b study. Lancet Neurol..

[54] Bigal M.E., Edvinsson L., Rapoport A.M., Lipton R.B., Spierings E.L., Diener H.C., Burstein R., Loupe P.S., Ma Y., Yang R. (2015). Safety, tolerability, and efficacy of TEV-48125 for preventive treatment of chronic migraine: A multicentre, randomised, double-blind, placebo-controlled, phase 2b study. Lancet Neurol..

[55] Dodick D.W., Ashina M., Brandes J.L., Kudrow D., Lanteri-Minet M., Osipova V., Palmer K., Picard H., Mikol D.D., Lenz R.A. (2018). ARISE: A phase 3 randomized trial of erenumab for episodic migraine. Cephalalgia.

[56] Dodick D.W., Goadsby P.J., Silberstein S.D., Lipton R.B., Olesen J., Ashina M., Wilks K., Kudrow D., Kroll R., Kohrman B. (2014). Safety and efficacy of ALD403, an antibody to calcitonin gene-related peptide, for the prevention of frequent episodic migraine: A randomised, double-blind, placebo-controlled, exploratory phase 2 trial. Lancet Neurol..

[57] Dodick D.W., Goadsby P.J., Spierings E.L., Scherer J.C., Sweeney S.P., Grayzel D.S. (2014). Safety and efficacy of LY2951742, a monoclonal antibody to calcitonin gene-related peptide, for the prevention of migraine: A phase 2, randomised, double-blind, placebo-controlled study. Lancet Neurol..

[58] Dodick D.W., Lipton R.B., Silberstein S., Goadsby P.J., Biondi D., Hirman J., Cady R., Smith J. (2019). Eptinezumab for prevention of chronic migraine: A randomized phase 2b clinical trial. Cephalalgia.

[59] Ferrari M.D., Diener H.C., Ning X., Galic M., Cohen J.M., Yang R., Mueller M., Ahn A.H., Schwartz Y.C., Grozinski-Wolff M. (2019). Fremanezumab versus placebo for migraine prevention in patients with documented failure to up to four migraine preventive medication classes (FOCUS): A randomised, double-blind, placebo-controlled, phase 3b trial. Lancet.

[60] Goadsby P.J., Reuter U., Hallström Y., Broessner G., Bonner J.H., Zhang F., Sapra S., Picard H., Mikol D.D., Lenz R.A. (2017). A controlled trial of erenumab for episodic migraine. N. Engl. J. Med..

[61] Goadsby P.J., Silberstein S.D., Yeung P.P., Cohen J.M., Ning X., Yang R., Dodick D.W. (2020). Long-term safety, tolerability, and efficacy of fremanezumab in migraine: A randomized study. Neurology.

[62] Hu B., Li G., Li X., Wu S., Yu T., Li X., Zhao H., Jia Z., Zhuang J., Yu S. (2022). Galcanezumab in episodic migraine: The phase 3, randomized, double-blind, placebo-controlled PERSIST study. J. Headache Pain.

[63] Lipton R.B., Goadsby P.J., Smith J., Schaeffler B.A., Biondi D.M., Hirman J., Pederson S., Allan B., Cady R. (2020). Efficacy and safety of eptinezumab in patients with chronic migraine: PROMISE-2. Neurology.

[64] Mulleners W.M., Kim B.K., Láinez M.J.A., Lanteri-Minet M., Pozo-Rosich P., Wang S., Tockhorn-Heidenreich A., Aurora S.K., Nichols R.M., Yunes-Medina L. (2020). Safety and efficacy of galcanezumab in patients for whom previous migraine preventive medication from two to four categories had failed (CONQUER): A multicentre, randomised, double-blind, placebo-controlled, phase 3b trial. Lancet Neurol..

[65] Reuter U., Ehrlich M., Gendolla A., Heinze A., Klatt J., Wen S., Hours-Zesiger P., Nickisch J., Sieder C., Hentschke C. (2022). Erenumab versus topiramate for the prevention of migraine–a randomised, double-blind, active-controlled phase 4 trial. Cephalalgia.

[66] Reuter U., Goadsby P.J., Lanteri-Minet M., Wen S., Hours-Zesiger P., Ferrari M.D., Klatt J. (2018). Efficacy and tolerability of erenumab in patients with episodic migraine in whom two-to-four previous preventive treatments were unsuccessful: A randomised, double-blind, placebo-controlled, phase 3b study. Lancet.

[67] Sakai F., Suzuki N., Kim B.K., Igarashi H., Hirata K., Takeshima T., Ning X., Shima T., Ishida M., Iba K. (2021). Efficacy and safety of fremanezumab for chronic migraine prevention: Multicenter, randomized, double-blind, placebo-controlled, parallel-group trial in Japanese and Korean patients. Headache J. Head Face Pain.

[68] Sakai F., Suzuki N., Kim B.K., Tatsuoka Y., Imai N., Ning X., Ishida M., Nagano K., Iba K., Kondo H. (2021). Efficacy and safety of fremanezumab for episodic migraine prevention: Multicenter, randomized, double-blind, placebo-controlled, parallel-group trial in Japanese and Korean patients. Headache J. Head Face Pain.

[69] Sakai F., Takeshima T., Tatsuoka Y., Hirata K., Lenz R., Wang Y., Cheng S., Hirama T., Mikol D.D. (2019). A randomized phase 2 study of erenumab for the prevention of episodic migraine in Japanese adults. Headache J. Head Face Pain.

[70] Skljarevski V., Matharu M., Millen B.A., Ossipov M.H., Kim B.-K., Yang J.Y. (2018). Efficacy and safety of galcanezumab for the prevention of episodic migraine: Results of the EVOLVE-2 phase 3 randomized controlled clinical trial. Cephalalgia.

[71] Skljarevski V., Oakes T.M., Zhang Q., Ferguson M.B., Martinez J., Camporeale A., Johnson K.W., Shan Q., Carter J., Schacht A. (2018). Effect of different doses of galcanezumab vs placebo for episodic migraine prevention: A randomized clinical trial. JAMA Neurol..

[72] Stauffer V.L., Dodick D.W., Zhang Q., Carter J.N., Ailani J., Conley R.R. (2018). Evaluation of galcanezumab for the prevention of episodic migraine: The EVOLVE-1 randomized clinical trial. JAMA Neurol..

[73] Sun H., Dodick D.W., Silberstein S., Goadsby P.J., Reuter U., Ashina M., Saper J., Cady R., Chon Y., Dietrich J. (2016). Safety and efficacy of AMG 334 for prevention of episodic migraine: A randomised, double-blind, placebo-controlled, phase 2 trial. Lancet Neurol..

[74] Takeshima T., Sakai F., Hirata K., Imai N., Matsumori Y., Yoshida R., Peng C., Cheng S., Mikol D.D. (2021). Erenumab treatment for migraine prevention in Japanese patients: Efficacy and safety results from a Phase 3, randomized, double-blind, placebo-controlled study. Headache J. Head Face Pain.

[75] Wang S.J., Roxas A.A., Saravia B., Kim B.K., Chowdhury D., Riachi N., Tai M.S., Tanprawate S., Ngoc T.T., Zhao Y.J. (2021). Randomised, controlled trial of erenumab for the prevention of episodic migraine in patients from Asia, the Middle East, and Latin America: The EMPOwER study. Cephalalgia.

[76] Winner P.K., McAllister P., Chakhava G., Ailani J., Ettrup A., Krog Josiassen M., Lindsten A., Mehta L., Cady R. (2021). Effects of intravenous eptinezumab vs placebo on headache pain and most bothersome symptom when initiated during a migraine attack: A randomized clinical trial. JAMA.

[77] Yu S., Kim B.K., Wang H., Zhou J., Wan Q., Yu T., Lian Y., Arkuszewski M., Ecochard L., Wen S. (2022). A phase 3, randomised, placebo-controlled study of erenumab for the prevention of chronic migraine in patients from Asia: The DRAGON study. J. Headache Pain.

[78] Labastida-Ramírez A., Caronna E., Gollion C., Stanyer E., Dapkute A., Braniste D., Naghshineh H., Meksa L., Chkhitunidze N., Gudadze T. (2023). Mode and site of action of therapies targeting CGRP signaling. J. Headache Pain.

[79] Concato J., Shah N., Horwitz R.I. (2000). Randomized, controlled trials, observational studies, and the hierarchy of research designs. N. Engl. J. Med..

[80] Diener H.C., Tassorelli C. (2020). Guidelines of the International Headache Society for controlled trials of preventive treatment of migraine attacks in episodic migraine in adults. Cephalalgia.

[81] van der Arend B.W., Verhagen I.E., van Leeuwen M., van der Arend M.Q., van Casteren D.S., Terwindt G.M. (2023). Defining migraine days, based on longitudinal E-diary data. Cephalalgia Int. J. Headache.

[82] Houle T.T., Turner D.P., Houle T.A., Smitherman T.A., Martin V., Penzien D.B., Lipton R.B. (2013). Rounding behavior in the reporting of headache frequency complicates headache chronification research. Headache J. Head Face Pain.

[83] Tassorelli C., Diener H.C., Dodick D.W., Silberstein S.D., Lipton R.B., Ashina M., Becker W.J., Ferrari M.D., Goadsby P.J., Pozo-Rosich P. (2018). Guidelines of the International Headache Society for controlled trials of preventive treatment of chronic migraine in adults. Cephalalgia.

